# Metal‐ and Solvent‐Free Transesterification and Aldol Condensation Reactions by a Homogenous Recyclable Basic Ionic Liquid Based on the 1,3,5‐Triazine Framework

**DOI:** 10.1002/open.202100091

**Published:** 2021-08-05

**Authors:** Yanqiu Hu, Mingqi Ren, Milad Kazemnejadi

**Affiliations:** ^1^ The Pharmaceutical College of Jiamusi University Jiamusi University 154007 Jiamusi China; ^2^ Department of Chemistry College of Science Shiraz University 7194684795 Shiraz Iran

**Keywords:** aldol condensation reactions, high basicity, ionic liquids, recyclability, transesterification reactions

## Abstract

A new recyclable basic ionic liquid was introduced as an efficient catalyst for aldol condensation and transesterification reactions under environmentally friendly conditions. The catalyst was prepared based on methyl imidazolium moieties bearing hydroxide counter anions *via* the Hofmann elimination on a 1,3,5‐triazine framework. The ionic liquid with two functionalities including anion stabilizer and high basicity, was used as an efficient catalyst for aldol condensation as well as transesterification reaction of a variety of alkyl benzoates. All reactions were performed in the absence of any external reagent, co‐catalyst, or solvent, in line with environmental protection. The kinetics isotope effect (KIE) was conducted for the transesterification reaction to elucidate the mechanism and rate determining step (RDS). It worth noted that, the homogeneous catalyst could be recycled from the reaction mixture and reused for several consecutive runs with insignificant drop of basicity and conversion.

## Graphical abstract

**A homogenous recyclable ionic liquid** with high basicity has been developed for metal‐ and solvent‐free aldol condensation and transesterification reaction.

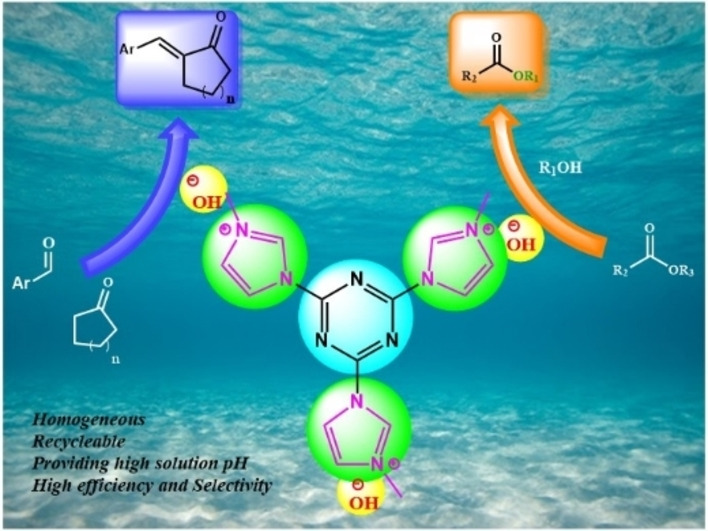

## 1 Introduction

Aldol condensation and transesterification, are two examples of the most practical reactions in industry, especially in the field of fuel and energy, which are usually catalyzed by a basic reagent in the presence of a catalyst.[^1^, ^2^, ^3^, ^4^] Aldol condensation has a wide range of application in fine chemicals synthesis; and includes two steps:^[5]^ (i) the intermediate formation of enols by a basic reagent, and (ii) the formation of a C−C bond between the enols and the carbonyl group that can be takes place *via* the reaction of an adsorbed molecule and a molecule in the fluid (Ely‐Rideal model) or occurs through a two‐molecular surface reaction (Langmuir‐Hinshelwood model) for heterogeneous catalysts.^[6]^ The next two steps of the re‐protonation and dehydration lead to the desired product. Various catalytic systems including homogenous and heterogeneous have been developed for these reactions, which can be point to alumina,^[5]^ zeolite,^[7]^ hydrotalcites,^[8]^ copolymer,^[1]^ TiO_2_, HAP, and MgO,^[2]^ catalysts.

Esters are the important class of chemicals that are widely used in agriculture, biological systems, polymers, as well as useful building blocks in organic synthesis.^[9]^ The transesterification reaction is also one of the basic reactions in organic chemistry and one of the most effective methods for the preparation of different types of esters, especially in industry.[^10^, ^11^] In addition, transesterification of triglycerides (in oils) with short‐chain alcohols such as methanol, ethanol, and propane leads to the production of biodiesel plant fuel.^[9]^ The transesterification reaction involves the exchange of an alcohol moiety in one ester with another alcoholic group in the presence of a catalyst.^[11]^ However, the two factors of (1) equilibrium nature and reversibility of the reaction and (2) the low reactivity of the ester functional group, are two limiting factors for achieving the desired selectivity/efficiency for this reaction.[^13^, ^14^]

Transesterification reaction is usually catalyzed by the protic and Lewis acid‐based catalysts, enzymes, antibodies, and bases. Recently, Tanaka *et al*. used an epoxide as a pre‐catalyst for metal‐free catalytic transesterification reaction.^[9]^ *N*‐heterocyclic carbine,^[13]^ organic methyl carbonate salts,[^14^, ^15^] maghemite‐ZnO,^[3]^ M_2_CO_3_ of MI (M=K, Na, Li, Cs),^[10]^ organic and inorganic bases,^[16]^ Lewis acids,^[12]^ and nano CuFe_2_O_4_ ^[11]^ were used as a catalyst for transesterification of esters.

**Scheme 1:**
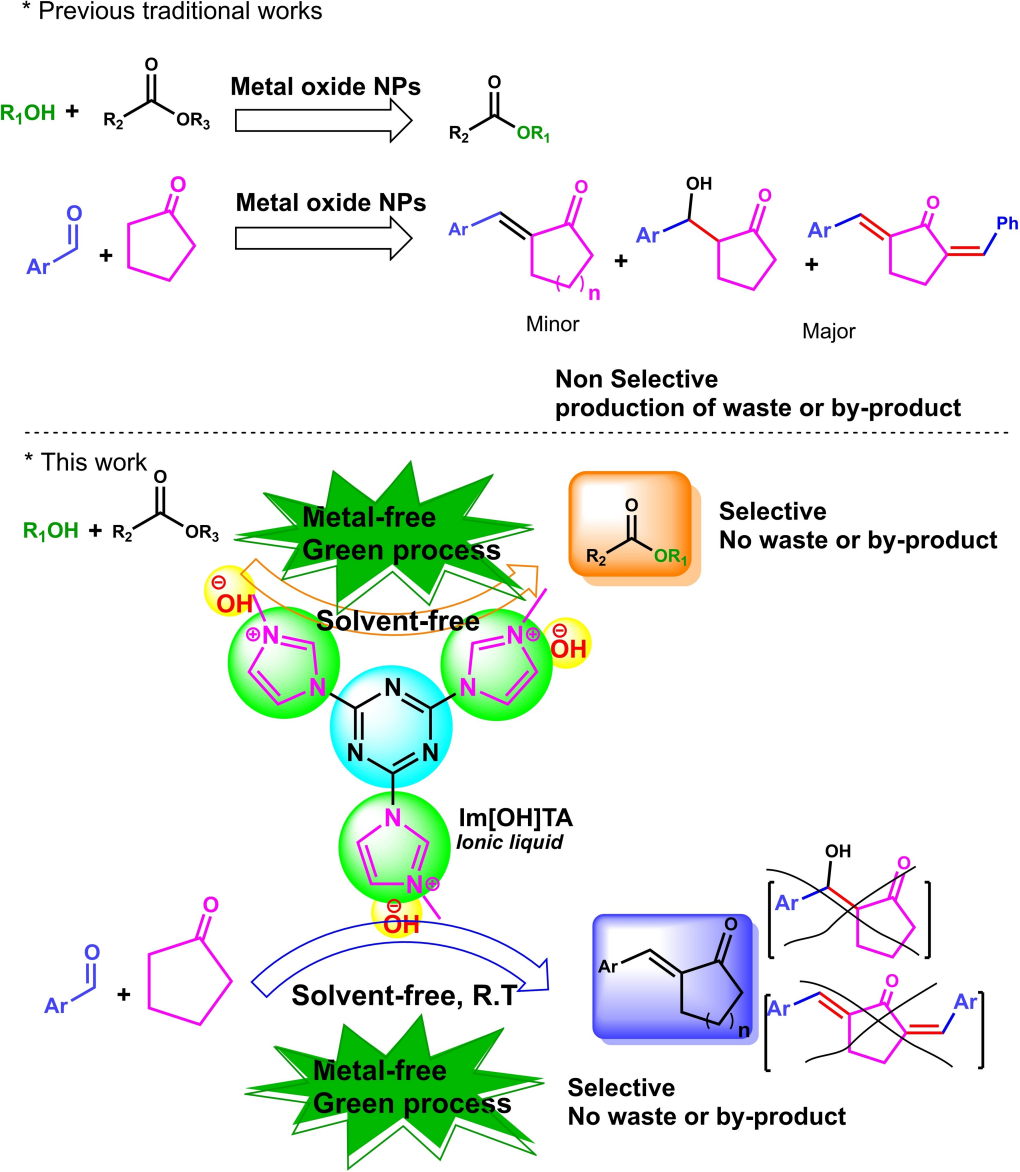
Selective and green transformation of aldol condensation and transesterification reaction catalyzed by [TAIm]OH ionic liquid.

Most of the methods introduced for these reactions include drawbacks such as: high reaction temperatures, non‐selectivity, lack of extensive consistency with most of substrates, lack of biocompatibility, high cost, use of toxic solvents, and toxic metal oxide NPs, which is not environmentally friendly (Scheme 1). The selectivity for aldol condensation is very important, as formation of aldol, acid and alcohol by‐products (due to Cannizzaro reaction) are possible (Scheme 1).[^9^, ^16^] Moreover, most of them is catalyzed by the non‐recyclable metal‐oxide NPs that provides un‐condensed aldol product or bis‐condensed product, as major product, causes destructive environmental effects (Scheme 1). Given the widespread application of transesterification and aldol condensation in industry (especially biofuel production), the introduction of a reliable and cost‐effective catalyst with a simple and sustainable design as well as environmentally friendly nature seems to be very necessary. Although heterogeneous catalysts have the advantage of recovering from the reaction medium than the homogenous type, their lower productivity than the homogenous type has led to them being used less in industries.^[17]^ In addition, discarding nano‐compounds leads to adverse environmental impacts.[^18^, ^19^] Therefore, the preparation and expansion of a recyclable catalyst with homogenous nature is a very useful strategy for catalysts used in industry. In addition, the deactivation of the catalyst in the reactor is one of the challenges facing the industry.^[20]^

Ionic liquids or molten salts are an interesting group of chemical compounds that are composed of organic cations and organic or mineral anions, but have completely different properties from the ionic salts.[^21^, ^22^] The properties of ionic liquids are highly dependent on the selection of cations and anions, and by changing them, a range of application of ionic liquids can be provided.^[23]^ Wide liquid range is one of the most important features that determines their use as an effective solvent.^[23]^ Most ionic liquids have good thermal and chemical stability and have low vapor pressure.[^21^, ^24^] Non‐flammability and non‐corrosive properties are also among the most common properties of ionic liquids.[^23^, ^25^] For this reason, they have been used as green solvents in most organic reactions, including hydrogenation, oxidation, coupling reactions, Diels‐alder reaction, etc.^[26]^ Application in extraction and separation (distillation extraction, gas separation),^[27]^ energy and clean technologies (color‐sensitive solar cells, supercapacitors, as electrolytes for batteries, etc.), recovery of useful substances in biomass, application in industries (as lubricants and additives, as surfactants, fuels, additives to paints and coatings), spectroscopy, electrochemistry (modification of electrode), chromatography (preparation of stationary phase),^[28]^ stability and protein and enzymes activity in the presence of ionic liquids, etc. are among the other uses of ionic liquids.[^21^, ^22^, ^23^, ^24^, ^25^, ^26^, ^27^, ^28^, ^29^, ^30^]

**Scheme 2:**
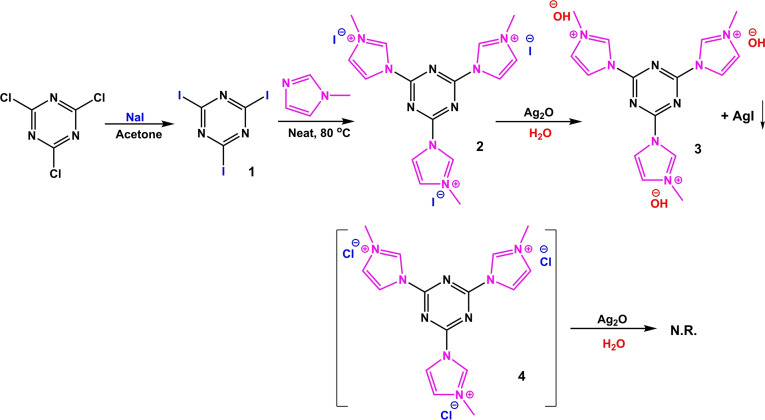
Preparation of imidazolium hydroxide‐triazine ([TAIm]OH) as a strong basic ionic liquid.

In this study, a homogeneous ionic liquid consists of [HO^−^] counter anions and imidazolium cations on a triazine framework was prepared and characterized (Scheme 2). The ionic liquid with the basic property provided high pHs of about 13–14 in solutions and subsequently provided a high catalytic activity for the transesterification as well as aldol condensation. It is also important to note that, unlike other homogenous catalysts reported in organic synthesis, the ionic liquid developed here is could be recycled, the products are easily isolated (simple work‐up), and there was no unwanted waste throughout the work‐up, and finally, high selectivity of products was achieved.

## 2 Results and Discussion

### 2.1 Catalyst Characterization

**Scheme 3:**
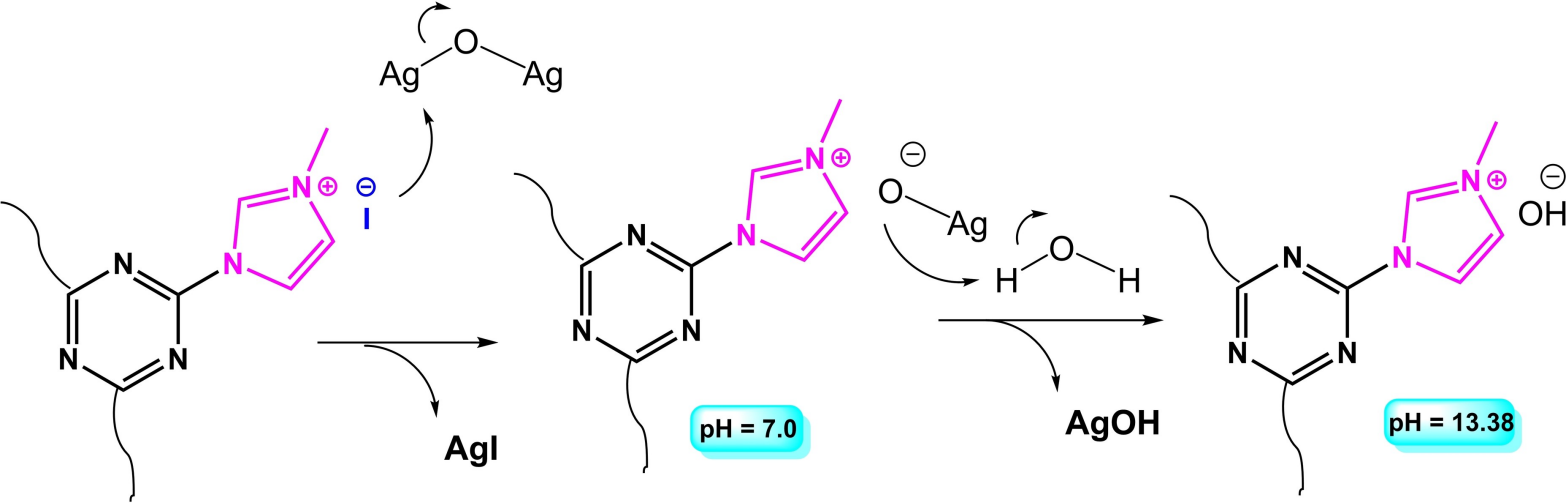
A mechanistic view for Hofmann elimination during the preparation of [TAIm]OH ionic liquid.

According to the acid titration assay, there is 10.0 mmol hydroxide anion in 1.0 mml of the ionic liquid. This gives a density equal to 1.55 g.cm^−3^ for the ionic liquid, which is completely in agreement with the measured density by a standard instrument (1.52 g.cm^−3^). Also, the viscosity of [TAIm]OH was found to be 1194 cP. Table 1 shows the general physical properties of [TAIm]OH. ^1^H‐NMR, ^13^C‐NMR and EDX analyses were completely confirmed the chemical structures of **2** and **3** compounds (Scheme 2), which all three Cl sites in 2,4,6‐trichloro‐1,3,5‐triazine (TCT) were functionalized with iodide, and subsequently with methyl imidazole. In addition, the complete elimination of the peak in the binding energies corresponds to iodide in the EDX spectrum of **3**, confirmed that the Hofmann elimination reaction was successful. Scheme 3 shows a mechanistic view for this transformation, where iodide counter ion attack to Ag_2_O and gives TAIm[OAg] intermediate. Then, in the presence of a water molecule, AgOH is formed and provides the desire product. Another prove for this process was the formation of AgI sediments, which characterized by FTIR analysis. It worth noted that the reaction was failed with TCT, and didn't found any product for **4** (Scheme 2). This could be directly related to the difference of atomic radius of Cl (175 pm) and iodide (198 pm), wherein Iodine binds more weakly to the imidazole cation than chlorine. However, with cyanuric iodide, the reaction processed efficiently.

**Table 1:** Physical properties of [TAIm]OH ionic liquid

Physical properties	Mw [g.mol^−1]^	Color/appearance	Density [g.cm^−3]^	Viscosity [cP]
Value	375.4	Yellow oil	1.52^[a]^	1194

[a] Based on apparatus. 1.55 g.cm^−3^ from the acid titration assay.

### 2.2 Reaction Optimization

The reaction between benzaldehyde and cyclopentanone was chosen to optimize and study the reaction parameters of the aldol condensation. Some of the results are summarized in Table 2. The optimal ratio between the three reactants including ionic liquid (as a basic catalyst), ketone and aldehyde was 2.5: 12: 1. In this ratio, the mixture has a pH of 13.84, which created 96 % conversion for the aldol condensation for 2 hours (Table 2, entry 4). The reaction time was significantly increased in the less molar ratios of the ionic liquid (Table 2, entry 3). In this ratio, the pH of the solution was also slightly reduced and some aldol by‐products were found. No change was observed in the higher molar ratios of the ionic liquid, although the pH also increased slightly (Table 2, entry 5). The optimal amount for cyclopentanone was found to be 12.0 mmol. The conversion decreased in smaller and higher amounts (Table 2, entries 1–3). Aldol condensation also produced the highest conversion at room temperature and decreased with increasing temperature (increasing time and decreasing conversion) (Table 2, entry 6). The aldol condensation is an exothermic reaction, so that with increasing temperature, the conversion of aldol condensation product decreases.^[8]^ However, no improvement in conversion was observed in an ice bath. Table 2, entry 4, shows the optimal conditions for the aldol condensation. It worth noted that no 2‐hydroxy or 2‐(benzylidene)‐5‐(benzylidene) cyclopentan‐1‐one by‐products were found in all experiments, and 2‐benzylidenecyclopentan‐1‐one was only product, that reflects the selectivity and high activity of [TAIm]OH. Cannizzaro reaction is one of the most possible reaction in a basic medium, however, with no observation of any benzoic acid or benzyl alcohol in the mixture, it reflects the high selectivity of the ionic liquid, which is completely consistence with environmental considerations.

**Table 2:** Investigations on effective parameters for the aldol condensation of benzaldehyde with cyclopentanone catalyzed by [TAIm]OH ionic liquid^[a]^

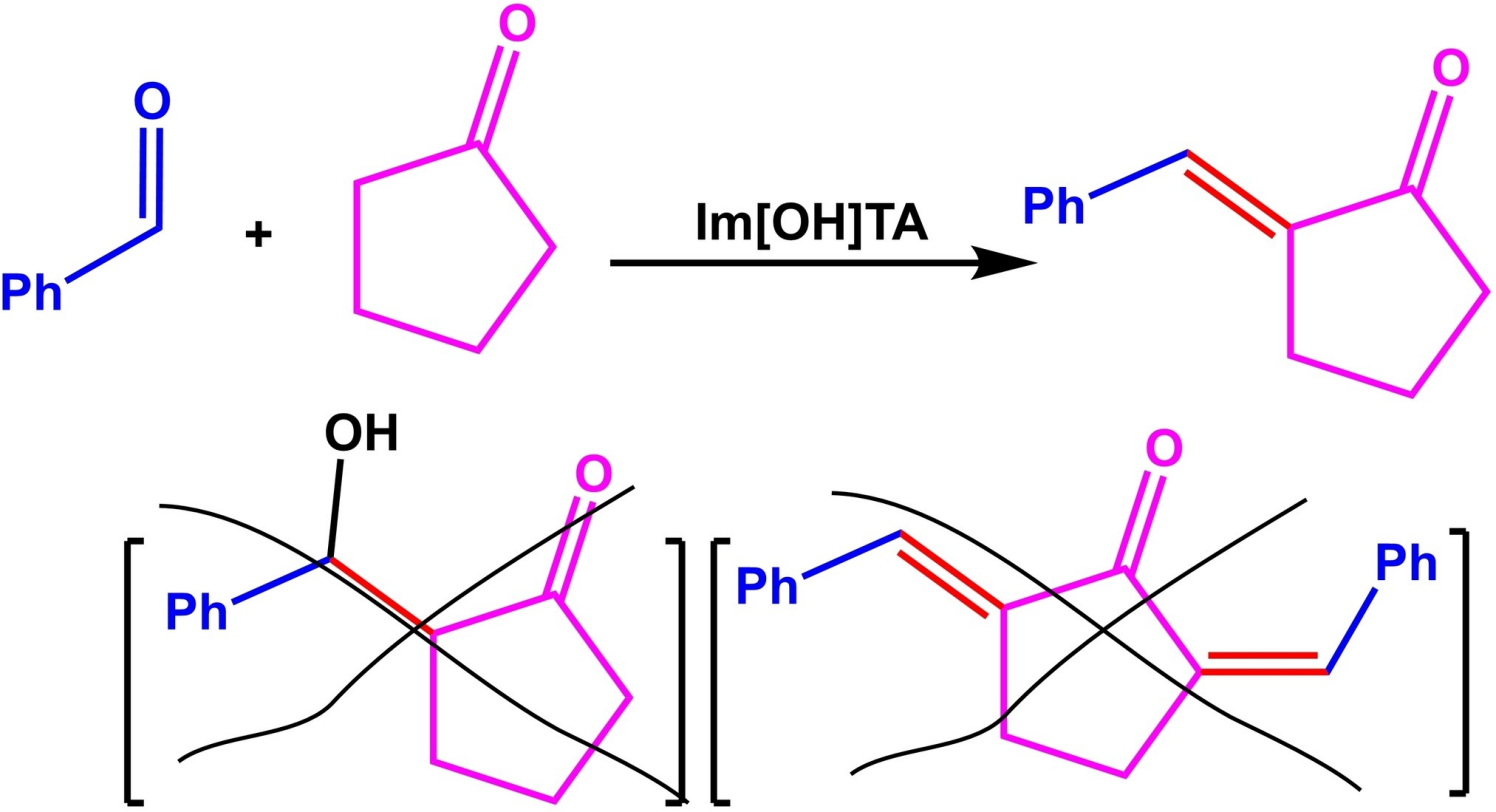					
Entry	Ionic liquid: ketone: aldehyde ratio (mmol)^[b,c]^	T [°C]	pH	Time [h]	Conversion [%]^[d]^
1	2.5: 10: 1	25	13.86	2.0	90
2	2.5: 14: 1	25	13.80	3.5	80
3^[e]^	2.0: 12: 1	25	13.75	3.0	90
4	2.5: 12: 1	25^[f]^	13.84	2.0	96
5	3.0: 12: 1	25	13.92	2.0	96
6	2.5: 12: 1	50	13.84	2.5	90

The reaction parameters were also optimized for the transesterification reaction of *n*‐butyl benzoate with methanol in the presence of [TAIm]OH ionic liquid as a model reaction. Firstly, the molar ratios between methanol, butyl benzoate and ionic liquid reactants were studied. As shown in Table 3, the highest conversion was obtained in twice the ratio of methanol and ionic liquid compared to butyl benzoate. The conversion in this ratio was 98 % for 5 h (pH 13.36). The conversion remained constant at higher values of ionic liquid (Table 3, entry 4), but at lower amounts, a significant reduction in conversion was observed.

**Table 3:** Investigations on effective parameters for the transesterification reaction of *n*‐butyl benzoate with methanol catalyzed by [TAIm]OH ionic liquid^[a]^

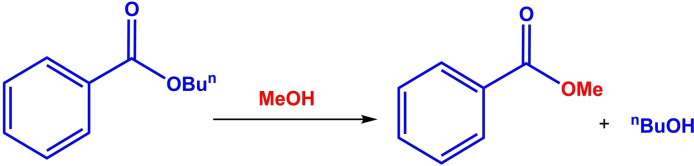					
Entry	Ionic liquid: butyl benzoate: MeOH (mmol)^[b]^	T [°C]	Time [h]	pH	Conversion [%]^[c]^
1	10: 5: 5	Reflux^[d]^	7	13.68	70
2	10: 5: 20	Reflux^[d]^	5	13.57	92
3	10: 5: 30	Reflux^[d]^	5	13.52	80
4	20: 5: 10	Reflux^[d]^	5	13.21	98
5	5: 5: 10	Reflux^[d]^	5	13.40	75
6	10: 5 : 10	Reflux^[d]^	5	13.36	98
7	10: 5: 10	40	10	13.37	60
8	10: 5: 10	R.T.	10	13.36	20

[a] Reaction conditions: *n*‐butyl benzoate, MeOH, ionic liquid, temperature, sealed tube. [b] mmol of ionic liquid is based on mmol of hydroxide ions in the ionic liquid. [c] GC conversion. [d] 65 °C.

The amount of methanol, as a solvent and as a reactant, had a significant effect on the conversion of the reaction, so that in the ratio equal to butyl benzoate, only 70 % of the conversion was observed for 7 hours (Table 3, Entry 1). In addition, the conversion was decreased with increase of MeOH to 4 times and 6 times than butyl benzoate amount (Table 3, Entry 2 and 3). The pH for all experiments was about 13.

### 2.3 Catalyst Activity

The catalytic activity of [TAIm]OH was then investigated for aldol condensation between aldehydes and cyclopentanone (or cyclohexane). The results of this study are given in Table 4. In general, good to excellent performance was achieved for all derivatives over a period of 2.0–4.5 hours.

**Table 4:** [TAIm]OH ionic liquid catalyzed aldol condensation reaction^[a]^

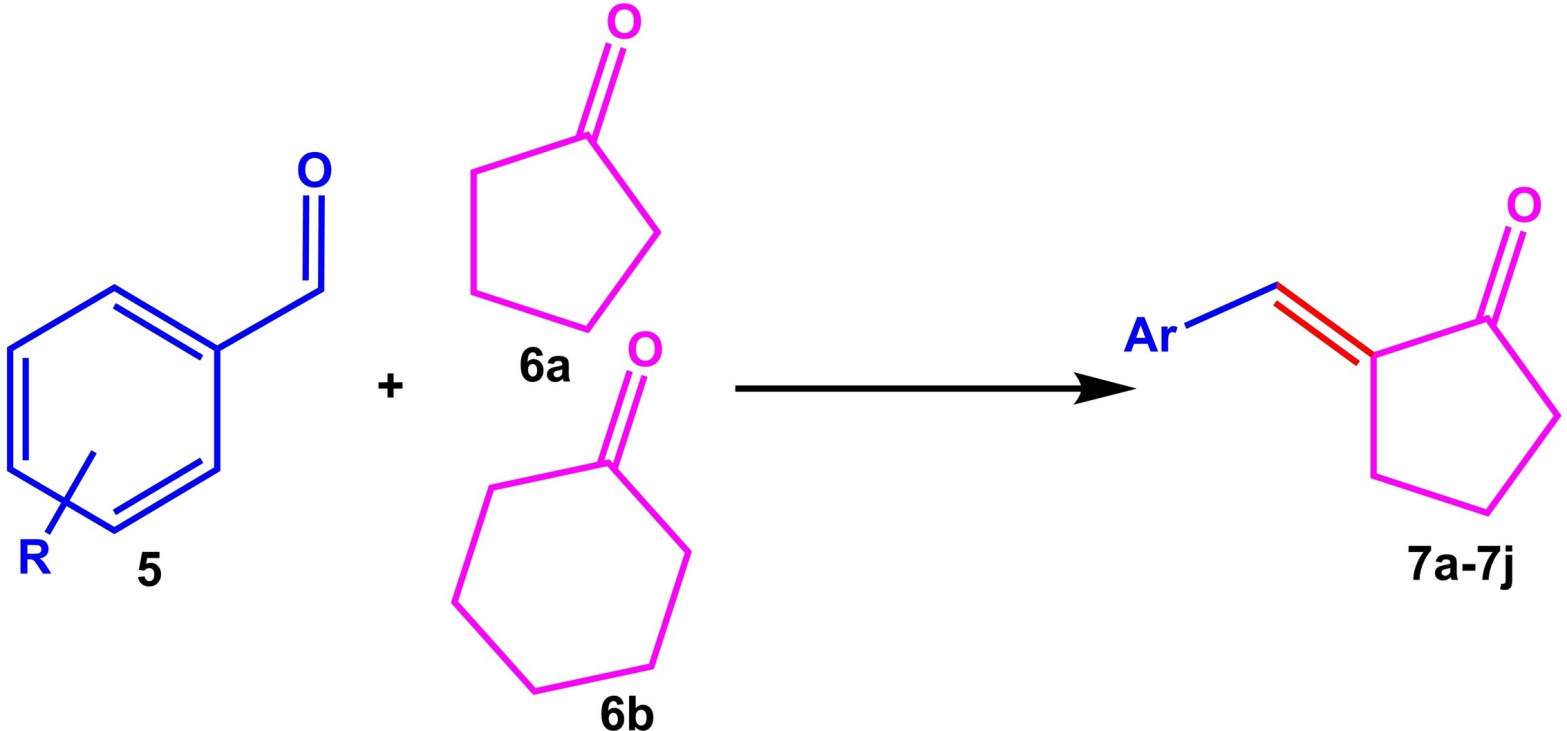					
Entry	R	Ketone	Product	Time (h)	Conversion [%]^[b]^
1	H	**6 a**	**7 a**	2	93
2	4‐MeO	**6 a**	**7 b**	3.5	90
3	4‐NO_2_	**6 a**	**7 c**	1.2	96
4	4‐OH	**6 a**	**7 d**	3	92
5	4‐Br	**6 a**	**7 e**	2.5	95
6	H	**6 b**	**7 f**	3	96
7	4‐MeO	**6 b**	**7 g**	3	85
8	4‐NO_2_	**6 b**	**7 h**	2.5	95
9	4‐OH	**6 b**	**7 i**	4	90
10	4‐Br	**6 b**	**7 j**	4.5	92

[a] Reaction conditions: Aldehyde (1.0 mmol), ketone (12.0 mmol), ionic liquid (0.2 ml, 2.5 mmol OH group), room temperature, sealed tube. [b] GC analysis.

Aldol condensations were obtained for cyclopentanone in shorter times than cyclohexanone, while almost similar conversions were obtained. The electronic effects (substitution on the aromatic ring) on conversion were completely noticeable in the reaction, so that electron withdrawing groups like NO_2_ produced better conversion than electron donor groups like OMe (for example, compare **7 b** with **7 c**, and **7 g** with **7 h**). Also, the catalytic activity of [TAIm]OH ionic liquid was evaluated towards transesterification of a variety of alkyl benzoates with different alcohols. Table 5 shows the results of the methyl‐, ethyl‐, propyl‐, and butyl‐benzoate transesterification with methanol, ethanol, and propanol alcohols. Satisfactory results were achieved for all alcohols.

**Table 5:** [TAIm]OH ionic liquid catalyzed transesterification reaction^[a]^

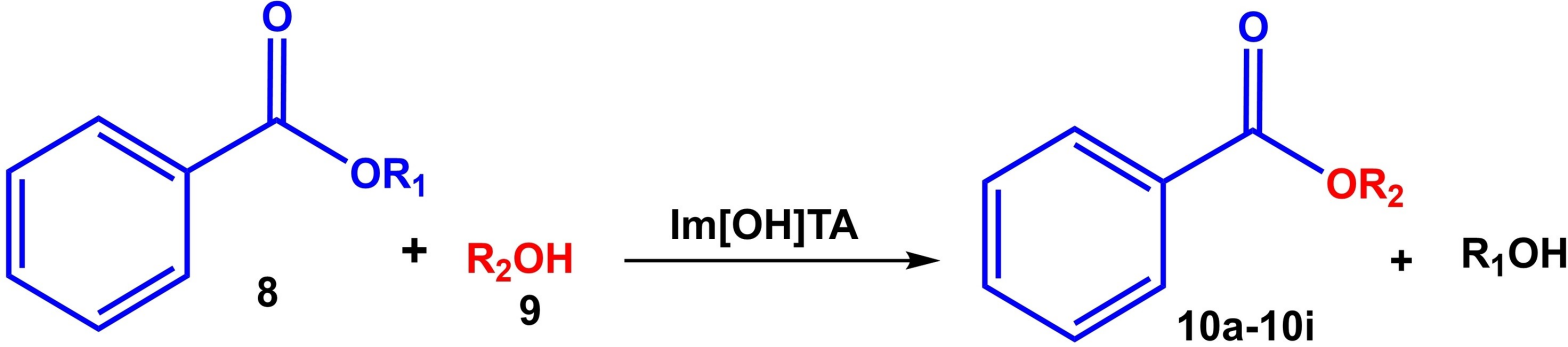					
Entry	R_1_	R_2_	Product	Time [h]	Conversion [%]^[b]^
1	Et	Me	**10 a**	14	75
2	*n*‐Pr	Me	**10 b**	9	80
3	*n*‐Bu	Me	**10 c**	5	98
4	Me	EtOH	**10 d**	10	80
5	*n*‐Pr	EtOH	**10 e**	12	84
6	*n*‐Bu	EtOH	**10 f**	12	85
7	Me	*n*‐Pr	**10 g**	7	77
8	Et	*n*‐Pr	**10 h**	5	95
9	*n*‐Bu	*n*‐Pr	**10 i**	4	96

[a] Reaction conditions: Benzoate ester (5.0 mmol), alcohol (10 mmol), ionic liquid (1.0 ml, 10.0 mmol OH group), sealed tube. The all reactions were performed under boiling points of alcohols. [b] GC analysis.

*n*‐Butyl benzoate provides higher conversion for all three alcohols than other benzoates (Table 5, entries 3,6,9). The conversion for ethanol was lower than for other alcohols, so that the reaction time was significantly different from the other two alcohols.

**Figure 1:**
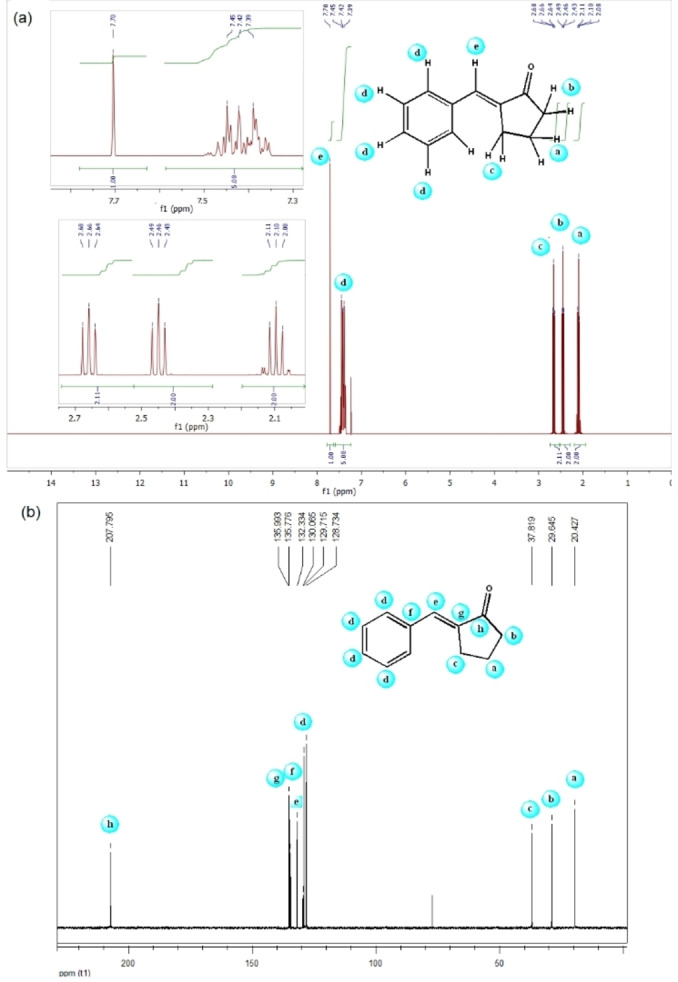
(a) ^1^H‐NMR (250 MHz) and (b) ^13^C‐NMR (62.9 MHz) spectra of **7 a** in CDCl_3_.

Figure 1 shows the NMR spectra of compound **7 a**. The protons of the cyclopentanone moiety ring appear in the region 2.10–2.66 ppm (Figure 1a). A series of peaks appearing with a center of 7.42 ppm were assigned to benzene ring protons. Vinyl protons are also found in the 7.70 ppm. The carbon spectrum also well confirms the structure of **7 a** with the presence of characteristic peaks related to aliphatic carbons, carbonyl and vinyl groups in (20.4–27.8), 207.7 and (132.3 and 135.7), respectively (Figure 1b).

Given that there are not many reports of catalytic aldol condensation reactions with ionic liquids such as tetramethylguanidine‐based ionic liquids,^[31]^ alanine‐supported protic ionic liquids,^[32]^ Brønsted ionic liquids,^[33]^ and ionic liquid supported organocatalyst of pyrrolidine amide;^[34]^ the results show an improvement in the aldol condensation catalyzed with ionic liquids (especially basic ionic liquids). Most ionic liquid‐based protocols are non‐recyclable or require high temperature, expensive and toxic reagents, and takes long times, and water removal requires separate heating in a further step. In addition, most of them do not have the basic property, and therefore their use is limited for other practical purposes. In this work, using [TAIm]OH, the aldol condensation reaction is performed at room temperature without the presence of any solvent or other additives. Another advantage of the present method is that it is directly leads to the condensation product by removing water (and creating a double bond). In addition, the basic property of the ionic liquid has led to its use as an effective catalyst in transesterification, which is one of the most important synthetic reactions in biodiesel production.

### 2.4 Reaction Mechanism

**Scheme 4:**
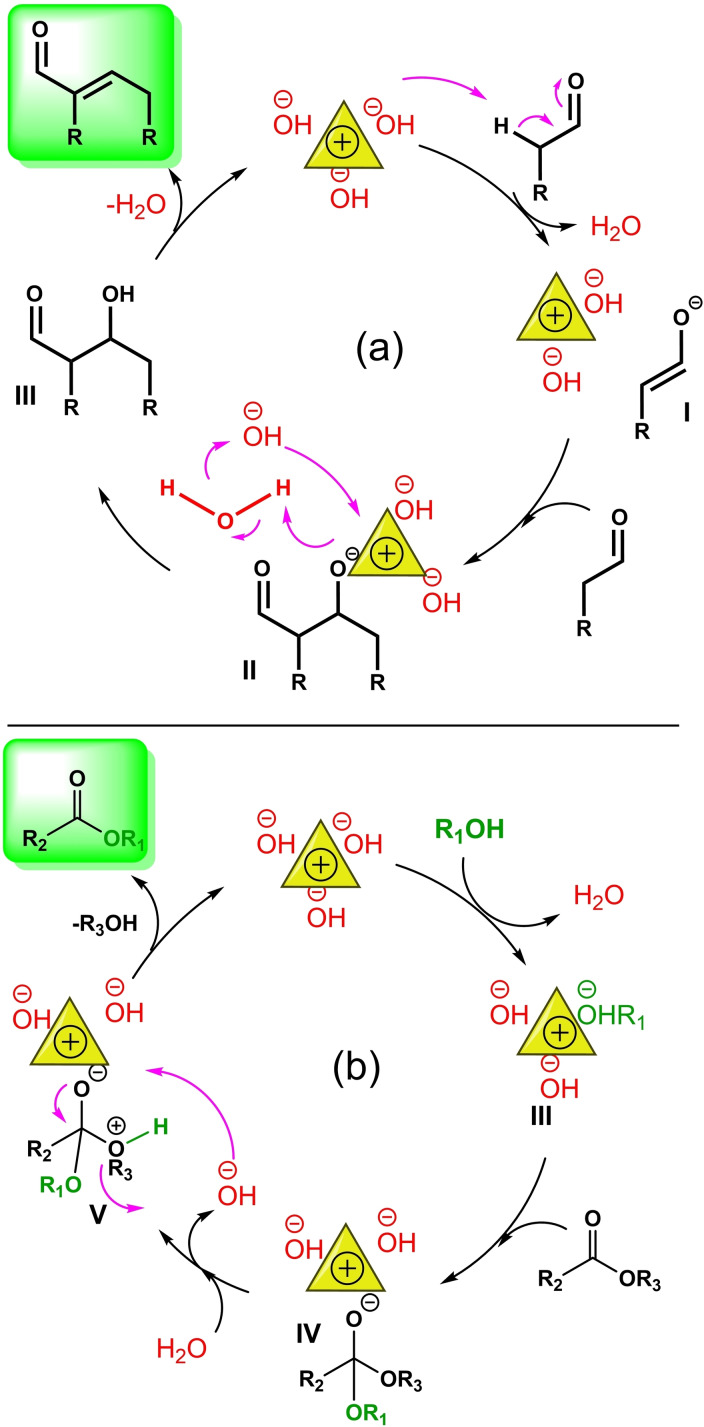
The plausible reaction mechanisms for (a) aldol condensation and (b) transesterification reaction catalyzed by [TAIm]OH.

**Scheme 5:**
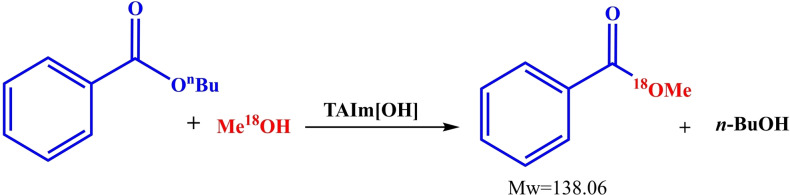
sotope effect study over the transesterification reaction of *n*‐butyl benzoate with Me^18^OH catalyzed by [TAIm]OH.

**Figure 2:**
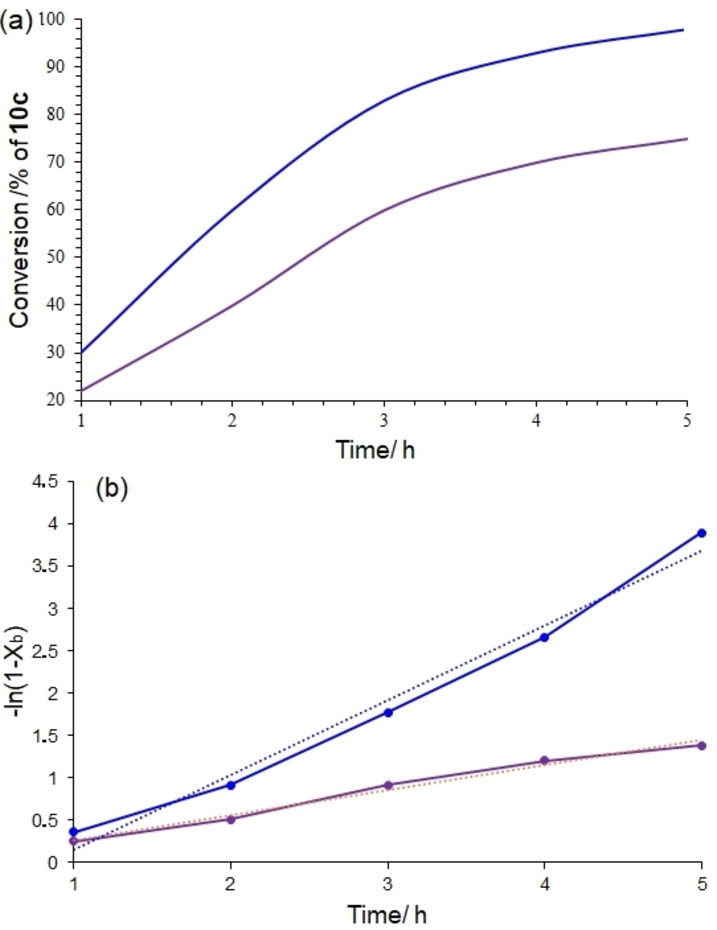
(a) Comparison between the kinetics of transesterification of *n*‐butyl benzoate with Me^18^OH (purple curves) with normal MeOH (blue curves) catalyzed by [TAIm]OH. The slopes of the lines correspond to the rate constant.

Two plausible mechanisms have been proposed for aldol condensation and transesterification reactions catalyzed by [TAIm]OH. Due to the presence of hydroxide groups in the ionic liquid, ketone is first converted to the enolate form (Scheme 4a, intermediate I) by the hydroxide counter ion groups in [TAIm]OH, which can be stabilized by the imidazolium groups in the ionic liquid. The next step is the nucleophilic attack of enolate to the carbonyl group of aldehyde, creating intermediate II (Scheme 4a), which, by abstracting protons from a water molecule, creates the desired aldol condensation product after removing a water molecule. Then, the catalyst by receiving a hydroxide group from H_2_O molecules formed by the transfer of protons to the aldol intermediate (Scheme 4a, intermediate II), goes back to the cycle. Simply, the transesterification reaction also begins with the abstracting of proton from alcohol by [TAIm]OH and the formation of intermediate III. The alkoxide group is also stabilized by the catalyst as a stable nucleophile. In the next step, the ester group is attacked by the alkoxide and creates intermediate IV. The presence of water causes the protonation of alcoholic group of ester, which is subsequently removed to form the desired ester product with the new alcoholic part. The catalyst also returns to the cycle after receiving an OH molecule from H_2_O molecule formed by the transfer of protons to intermediate V (Scheme 4b). This was proved by isotope labeling experiment using the reaction between CH_3_^18^OH with *n*‐butyl benzoate in the same reaction conditions described before to identify which bond of MeOH or *n*‐butyl benzoate broke (Scheme 5). The results from GC‐MS analysis clearly showed the major product with ^18^O. However, the conversion in this case was slightly decreased to 75 % (based on GC). The results suggest that Im−^18^O or ^18^O−H cleavage is the rate‐determining step. In this way, the conversion of transesterification (*X_b_*) was obtained from GC injection of the extracted product, and a linear relationship was provided between −ln(1‐*X_b_*) and the reaction time in agreement with the literature.[^35^, ^36^] Also, the kinetics was obtained for normal MeOH (Figure 2a).

The plot of −ln(1‐*X_b_*) versus the reaction time t creates the straight lines for both CH_3_^18^OH and normal CH_3_^16^OH according to the equation of first‐order reaction (Figure 2a): −ln(1‐*X_b_*)=*kt* in which k is the rate constant for the reaction. The rate constant for Me^16^OH was calculated as *k_16_* =4.9×10^−5^ s^−1^, and for Me^18^OH, *k_18_* =1.6×10^−5^ s^−1^. So, KIE=*k_16_*/*k_18_* =3.06. The results revealed that the bond dissociation of ^18^O−H or C−^18^O was the rate determining step and the hydroxide group in the newly prepared ester is comes from the alcohol used completely in agreement with the proposed mechanism (Figure 2b).

### 2.5 Control Experiments

To show that the catalytic activity of [TAIm]OH is unique, some control experiments were designed using [TAIm]I, TCT, NaOH, Ag_2_O, and [bmim]OH as a catalyst for the reactions of (i) benzaldehyde with cyclopentanone (preparation of **7 a**), and (2) transesterification reaction of *n*‐*n*‐butyl benzoate with methanol (preparation of **10 c**). Table 6 shows the results. Based on the results, [TAIm]I give 30 % and 10 % conversion for **7 a** and **10 c**, respectively at higher reaction times (Table 6, entry 1). TCT, NaOH, and Ag_2_O didn't show any detectable product under these conditions (Table 6, entries 2–4), and the results was as same as catalyst‐free conditions with zero conversion. Finally, the activity of [TAIm]OH was also compared with a commercially well‐known ionic liquid, 1‐butyl‐3‐methylimidazolium hydroxide, [bmim]OH (Table 6, entry 6), which provides 80 % and 45 % conversion for **7 a** and **10 c**, respectively (Table 6, entry 6). The results clearly proved the superiority of [TAIm]OH over all other component, and the observed catalytic activity for [TAIm]OH is unique.

**Table 6:** Control experiments for aldol condensation and transesterification reactions catalyzed by various compounds

Entry	Catalyst	Time [h]		Conversion [%]^[a]^	
		**7 a** ^[b]^	**10 c** ^[c]^	**7 a** ^[b]^	**10 c** ^[c]^
1	[TAIm]I^[d]^	4	7	30	10
2	TCT	2	5	–	–
3	NaOH_(aq)_ 10 %wt	2	5	3	–
4	Ag_2_O	2	5	–	–
5	–	2	5	–	–
6	[bmim]OH^[d]^	4	7	80	45

[a] GC analysis. [b] Reaction conditions: benzaldehyde (1.0 mmol), cyclopentanone (12.0 mmol), catalyst (2.5 mmol), room temperature, sealed tube. [c] Reaction conditions: *n*‐butyl benzoate (5.0 mmol), MeOH (10 mmol), catalyst (10.0 mmol), reflux, sealed tube. [d] 0.2 mL.

### 2.6 Recyclability of [TAIm]OH

**Figure 3:**
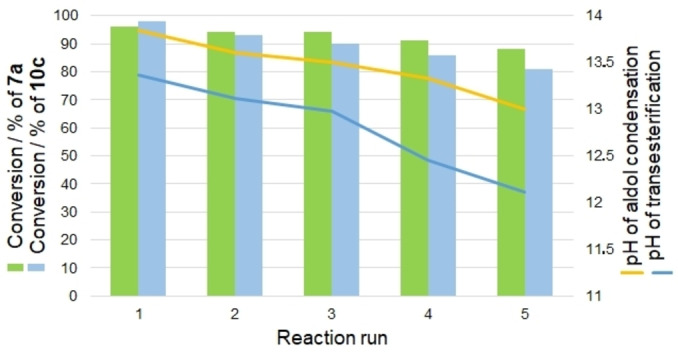
Recycling studies of [TAIm]OH over the catalytic reaction of aldol condensation and transesterification.

Recoverability and reusability of the homogeneous ionic liquid was studied over the aldol condensation as well as transesterification reactions. Deactivation and reactivity loss of ionic liquids is a common issue in organic synthesis, which affects the conversion of the product from environmental and economic viewpoints[^21^, ^23^] In this way, the recyclability of the ionic liquid was evaluated in (i) aldol condensation of benzaldehyde with cyclopentanone and (ii) transesterification reaction of *n*‐butyl benzoate with MeOH. It worth noted that, in each recycle, the pH of the solution was recorded. Figure 3 shows the results for 5 consecutive runs. The prominent advantage of the work was insignificant activity loss of the ionic liquid, which could be auto‐reactivated arising from regeneration of hydroxide ion in the ionic liquid during the reaction. This results were reflected in the proposed mechanism, where the ionic liquid could be simple preserve its hydroxide groups *via* regeneration of hydroxide ions during the reaction and subsequently showed high catalytic activity. As shown in Figure 3, there is not any considerable change in pH of the solution; and the observed small loss of activity may be related to loss of ionic liquid during work‐up in each cycle, which is inevitable. Furthermore, based on the titration assay of the recovered [TAIm]OH over the model aldol condensation reaction, there is 10 mmol OH− per mL of ionic liquid (mean of three repeats: 9.84, 9.65, 9.75). The complete results are in accordance with the reproduction of OH groups on the ionic liquid according to the proposed mechanism, which reflects the high stability of the catalyst, which can maintain its catalytic activity in successive cycles.

However, for the transesterification reaction, the activity loss was higher, and more decrease in pH was observed. This difference could be directly related to the harsher reaction conditions for the transesterification than the aldol condensation. The reaction profile is clean, so there is not any impurity responsible for the catalyst deactivation (blocking basic sites). Basic sites in [TAIm]OH are OH counter ions and regenerates during the reactions. Insignificant loss in catalytic activity of the ionic liquid can be related to the absence of any metal (generally transition metal) in the catalyst, as it is common in most metal‐based catalysts with significant inevitable metal leaching.[^21^, ^23^]

**Figure 4:**
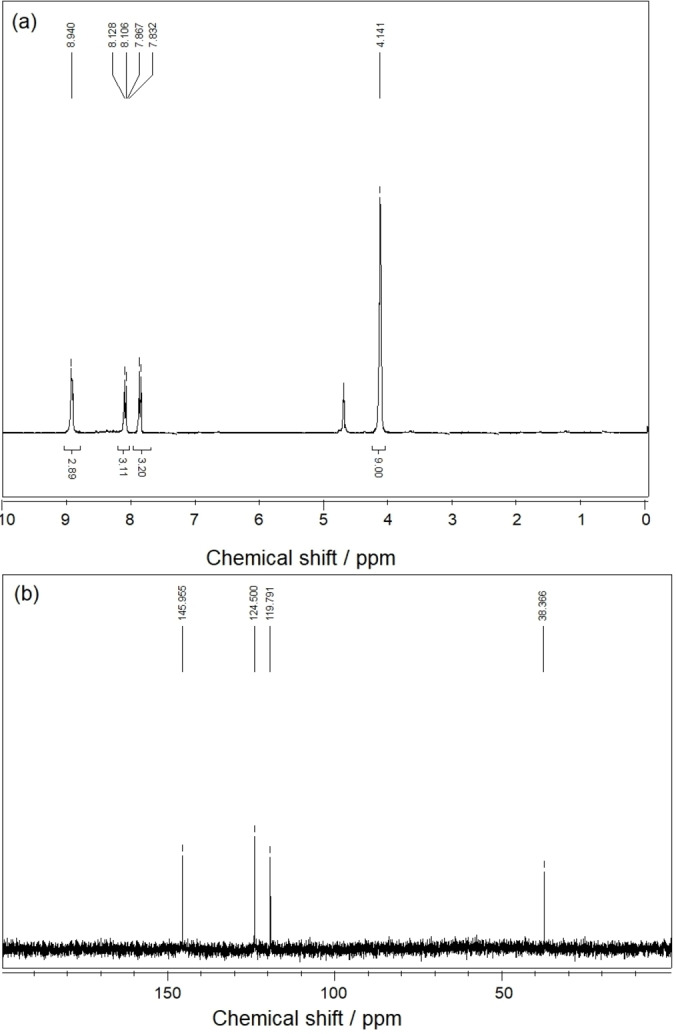
(a) ^1^H‐NMR and (b) ^13^C‐NMR spectra of the recovered [TAIm]OH after 5th over the preparation of **7 a**.

Finally, in order to investigate the structure of the catalyst during successive catalytic cycles, the recovered catalyst after the fifth cycle in the aldol condensation reaction (condensation of benzaldehyde with cyclopentanone, **7 a**) was characterized by NMR analysis. Figure 4 shows the ^1^H‐NMR and ^13^C‐NMR spectra from the recovered [TAIm]OH ionic liquid, which are exactly the same as the spectra from the freshly prepared catalyst. The result indicates the high stability of the catalyst, which is responsible for maintaining its catalytic activity during successive cycles.

## 3 Conclusions

In summary, we have developed a homogeneous recyclable basic ionic liquid ([TAIm]OH) with high catalytic activity as an anion stabilizer for efficient aldol condensation and transesterification reactions. [TAIm]OH plays three roles including: (i) providing high basic medium (high pHs) for the base‐catalyzed reactions, and (ii) stabilization of anions intermediates formed during the reactions, which acts as a driving force for the reactions, and (iii) acts as a strong solvent for the organic reactions. All the reactions were performed *via* a green process, i. e. at room temperature in the absence of any external base, additive, transition metal catalyst, or solvent. Moderate to high, and excellent conversions in some cases, were achieved for all of entries. High selectivity towards the desired aldol condensation and transesterification products was also another advantage of the ionic liquid. The highlight point of the work was recyclability of the homogeneous ionic liquid, which was successfully recovered and reused for at least 5 consecutive runs with a negligible loss of activity. KIE studies revealed the mechanism as well as RDS for the transesterification reaction. The results demonstrated the higher catalytic activity of [TAIm]OH towards the well‐known basic ionic liquid, [bmim]OH, over the both aldol condensation and transesterification reactions.

## Experimental Section

### Materials and Instrumentation

All chemical and reagents were provided in analytical grade from Sigma and Merck suppliers and used as received without any further purification. All solvents (with analytical grade) were dried before use. Metahnol‐^18^O 95 atom% for isotope labeling was provided from Sigma. 1‐Butyl‐3‐methylimidazolium hydroxide [bmim]OH (Form comparative analyses) was provided from Zhonglan Industry Co., Ltd. Supplier. Benzaldehyde was kept over molecular sieves in order to trap any possible traces of benzoic acid. The progress of reactions was monitored by thin layer chromatography (TLC) on silica gel (polygram SILG/UV 254 plates) or gas chromatography (GC) using a Shimadzu‐14B gas chromatography equipped with HP‐1 capillary column and N_2_ as carrier gas and anisole as an internal standard. FTIR spectra were taken on a JASCO FT/IR 4600 spectrophotometer using KBr disk. The ^1^H‐NMR (250 MHz) and ^13^C‐NMR (62.9 MHz) spectra were recorded on a Bruker Avance DPX‐250 spectrometer in D_2_O (for ionic liquids 2, 3), CDCl_3_, and DMSO‐*d_6_* solvents, and TMS as an internal standard. Elemental analysis was performed by energy dispersive X‐ray spectroscopy (EDX) method using a field emission scanning electron microscope, FESEM, JEOL 7600F, equipped with a spectrometer of energy dispersion of X‐ray from Oxford instruments. The pH of the solutions was measured by a OAKTON Lab Model Digital pH/ion meter instrument at room temperature. The viscosity of the ionic liquid was measured using a Thermo Scientific™ HAAKE™ Viscotester™ apparatus at room temperature.

### Preparation of 1,1′,1′′‐(1,3,5‐triazine‐2,4,6‐triyl)tris(3‐methyl‐1*H*‐imidazol‐3‐ium) Hydroxide ([TAIm]OH) (3)

In first, for the preparation of cyanuric iodide (1), cyanuric chloride (or 2,4,6‐Trichloro‐1,3,5‐triazine (TCT), 0.37 g, 2.0 mmol) was added to 20 mL of dry acetone at room temperature. Then, NaI (1.5 g, 10 mmol) was added to the mixture. The flask was sealed and the mixture was stirred for 12 h. The color of the solution was gradually turns into yellow. Then, 1‐methylimidazole (15.0 mmol) was added to the mixture, and the resulting mixture was refluxed for 24 h. [TAIm]OH (2) was extracted to *n*‐BuOH (3×10 mL). The resulting organic phases were placed into a vacuum oven (65 °C) for 24 h. Exchange of iodide counter ions with hydroxide was preformed *via* the Hofmann elimination procedure using Ag_2_O. Ag_2_O was also prepared in this study according to a simple procedure described elsewhere.^[37]^ [TAIm]OH (2, 1.0 mL) was added to 20 mL of distilled water, then Ag_2_O (8.0 mmol, 1.8 g) was added to the mixture in one step. The reaction was performed under reflux conditions for 8 h. The sediment (AgI) was filtered and the product was extracted to *n*‐BuOH (3×10 mL). The resulting organic phases were dried into a vacuum oven (65 °C). Scheme 2 shows a schematic view for the preparation of [TAIm]OH.

Characterization data for *2*: Yellow oil; ^1^H NMR (D_2_O, 250 MHz): δ=4.50 (s, 9H, CH_3_), 7.32 (d, 3H, *J*=6.25 Hz, Im−H), 7.96 (d, 3H, *J*=6.25 Hz, Im−H), 8.98 (s, 3H, Im−H) ppm; ^13^C NMR (D_2_O, 62.9 MHz): δ=38.0, 117.7, 126.2, 144.6 ppm; EDX (%wt)=C 26.55, N 18.88, I 44.57.

Characterization data for 3: Yellow oil; ^1^H NMR (D_2_O, 250 MHz): δ=4.14 (s, 9H, CH_3_), 7.86 (d, 3H, *J*=7.00 Hz, Im−H), 8.10 (d, 3H, *J*=7.00 Hz, Im−H), 8.98 (s, 3H, Im−H); ^13^C NMR (D_2_O, 62.9 MHz): δ=38.7, 118.8, 126.6, 145.6 ppm; EDX (%wt)=C 49.33, N 35.76, O 14.91.

### Determination of the Hydroxide Group in [TAIm]OH

The hydroxide content in [TAIm]OH was measured by an acid titration assay. In this test, 1.0 mL of the ionic liquid was added to 50 mL of distilled water and stirred vigorously for 10 min (pH 13.88). The resulting solutions was titrated with acetic acid 1.0 M in the presence of phenolphthalein indicator at room temperature under air atmosphere. About 11.96 mL of acetic acid was consumed at the end point (pH 7.0). At the same time, a blank was also titrated and the total volume of acetic acid was recorded. Based on the experiment, 1.0 mL of ionic liquid contain 10.0 mmol hydroxide group. So, with assumption of three equivalent of ^−^OH ions in a mmol of [TAIm]OH, there are 4 mmol [TAIm]OH in a 1.0 mL of ionic liquid. Based on Mw of the ionic liquid, it can be concluded that the density is equal to 1.55 g.cm^−3^.

### Typical Procedure for Aldol Condensation in the Presence of [TAIm]OH

The ionic liquid (0.2 ml), and 12.0 mmol of cyclopentanone were mixed in a sealed tube and vigorously stirred for 10 min. Then, benzaldehyde (1.0 mmol) was added to the mixture and stirred at room temperature for an appropriate time with a continuously monitoring by TLC. After the completion of the reaction, 10 mL of water and 10 mL of CH_2_Cl_2_ was added to the mixture. The aqueous phase was further extracted to another 10 mL CH_2_Cl_2_. The organic and aqueous layers were separated, and the organic phase was dried over MgSO_4_, and the resulting aldol condensation product was obtained after removal of the solvent under reduced pressure followed by flash chromatography. The ionic liquid with high polarity is transferred to the aqueous phase, was recycled with remove of water under reduced pressure and drying over 3 Å‐molecular sieve for 8 h. After each recycles, the pH the ionic liquid was measured and the hydroxide amount was determined by titration.

Characterization data for 2‐Benzylidenecyclopentanone (7a): ^1^H NMR (CDCl_3_, 250 MHz): δ=2.08 (m, 2H, H4), 2.45 (t, *J*=7.9 Hz, 2H, H5), 2.66 (t, *J**=*** 7.2, 2H, H3), 7.42 (m, 5H, H2“, H3”), 7.70 (s, 1H, H1′) ppm; ^13^C NMR (62.9 MHz, CDCl_3_): δ=20.4, 29.6, 37.8, 128.7, 129.7, 130. 0, 132.3, 135.7, 135.9, 207.7 ppm.

Characterization data for 2‐(4‐nitrobenzylidene)cyclopentanone (7c): ^1^H NMR (250 MHz, CDCl_3_): δ=2.14 (s, 2H, H4), 2.53 (s, 2H, H5), 2.57 (s, 2H, H3), 7.46 (t, *J* **=**7.5 Hz, 1H, H1′), 7.97 (s, 1H, H3′′), 8.01 (d, *J* **=**7.5 Hz, 2H, H2′′) ppm; ^13^C‐NMR (CDCl_3_, 62.9 MHz): δ=20.9, 29.5, 37.3, 123.6, 129.6, 130.0, 139.4, 141.4, 147.5, 207.9 ppm.

Characterization data for 2‐Benzylidenecyclohexanone (7 f): ^1^H NMR (CDCl_3_, 250 MHz): δ=1.68–1.75 (m, 4H, H4), 2.42–2.52 (m, 4H, H5), 7.35–7.47 (m, 5H, H2′′, H3′′, H4′′), 7.81 (s,1H, H1′) ppm; ^13^C NMR (CDCl_3_, 62.9 MHz): δ=23.5, 24.0, 28.2, 40.1, 128.2, 128.5, 130.3, 135.5, 135.7, 136.7, 200.5 ppm.

Characterization data for 2‐(4‐Nitrobenzylidene)cyclohexanone (7 h): ^1^H NMR (CDCl_3_, 250 MHz): δ=1.73 (m, 4H, H4), 2.52–2.63 (m, 4H, H3, H5, H6), 7.46 (d, *J* **=**7.5 Hz, 2H, H1′), 8.01 (d, J**=**7.5 Hz, 2H, H2′′), 8.40 (s, 1H, H3′′) ppm; ^13^C NMR (CDCl_3_, 62.9 MHz): δ=23.4, 23.7, 29.3, 40.4, 123.7, 130.0, 132.0, 140.5, 142.4, 147.6, 201.4 ppm.

### Typical Procedure for Transesterification Reactions in the Presence of [TAIm]OH

Typically, in a 15 mL sealed tube, ionic liquid (1.0 ml, containing 10.0 mmol OH group), methanol (10.0 mmol), and *n*‐butyl benzoate (5.0 mmol) were added. The tube was placed in a ChemiStation instrument, while its temperature was adjusted to boiling point of MeOH. The mixture was magnetically and vigorously stirred for an appropriate time, which monitored by GC analysis in various time intervals. Anisole was used as an internal standard and the pure analytical grade of the solvents was used for the quantitative GC measurements. The conversion and selectivity of the resulting benzoate was calculated by the following formula (1), 2:

(1) $$\mathrm{Conversion}(\mathrm{mol}\%)=\frac{(\mathrm{initial}\mathrm{mol}\%)-(\mathrm{final}\mathrm{mol}\%)}{\mathrm{initial}\mathrm{mol}\%}\times 100$$

(2) $$\mathrm{Selectivity}=\frac{\mathrm{GC}\mathrm{peak}\mathrm{area}\mathrm{of}\mathrm{desired}\mathrm{product}}{\mathrm{GC}\mathrm{peak}\mathrm{area}\mathrm{of}\mathrm{all}\mathrm{products}}\times 100$$

To recover the ionic liquid, 10 ml distilled water along with 10 ml *n*‐BuOH was added to the mixture. The organic and aqueous layers were separated, then, the solvent was removed from aqueous phase (containing ionic liquid) under reduced pressure and drying over 3 Å‐molecular sieve for 8 h. All other transesterification reaction was performed same as the present procedure, but the reaction temperature was adjusted to boiling point of each alcohol used for the reaction.

## Conflict of interest

The authors declare no conflict of interest.

## Supporting information

As a service to our authors and readers, this journal provides supporting information supplied by the authors. Such materials are peer reviewed and may be re‐organized for online delivery, but are not copy‐edited or typeset. Technical support issues arising from supporting information (other than missing files) should be addressed to the authors.

Supporting Information
