# Supplementary material for: Metal‐ and Solvent‐Free Transesterification and Aldol Condensation Reactions by a Homogenous Recyclable Basic Ionic Liquid Based on the 1,3,5‐Triazine Framework
Source: ChemistryOpen. 2021 Aug 5;10(8):775–83. doi: 10.1002/open.202100091 (PMC8340073; doi:10.1002/open.202100091)
Supplement: Supplementary file 1 — Supporting Information [file OPEN-10-775-s001.pdf]

ChemistryOpen

Supporting Information

Metal- and Solvent-Free Transesterification and Aldol Condensation Reactions by a Homogenous Recyclable Basic Ionic Liquid Based on the 1,3,5-Triazine Framework

Yanqiu Hu,* Mingqi Ren, and Milad Kazemnejadi*

Supporting Information

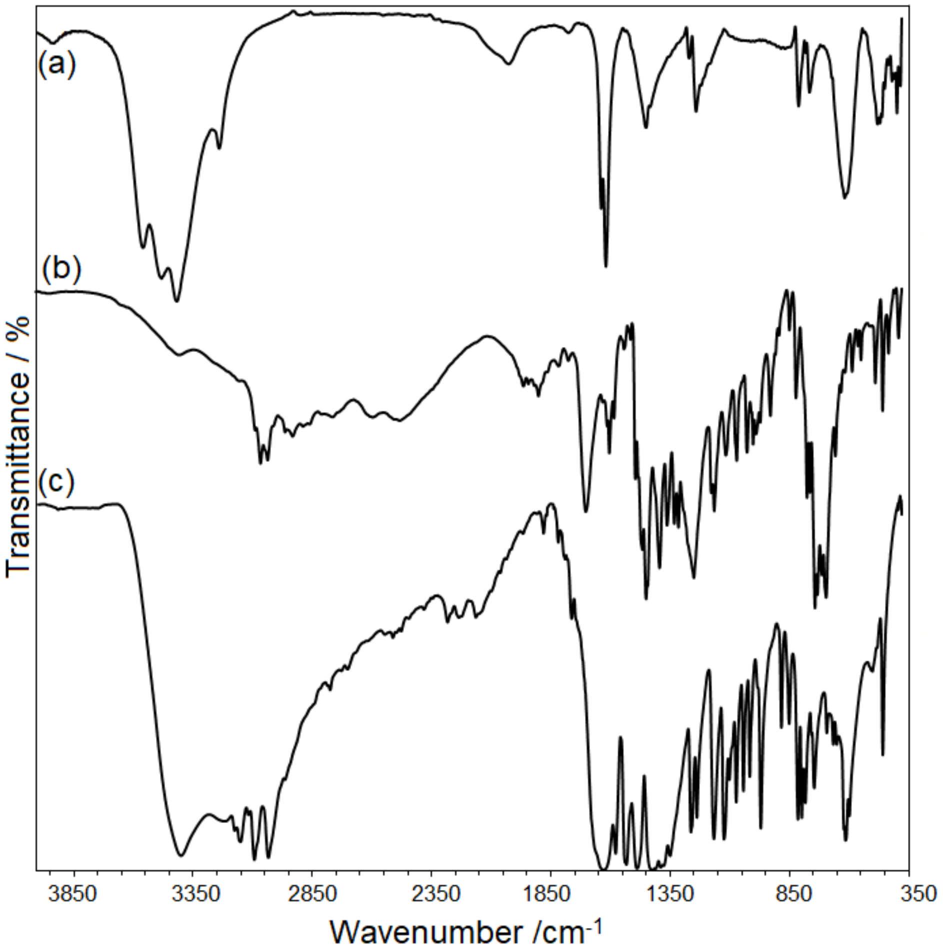

Fig. S1 FTIR spectra of (a) cyanuric iodide **1**, (b) Im[I]TA **2**, and (c) Im[OH]TA **3**

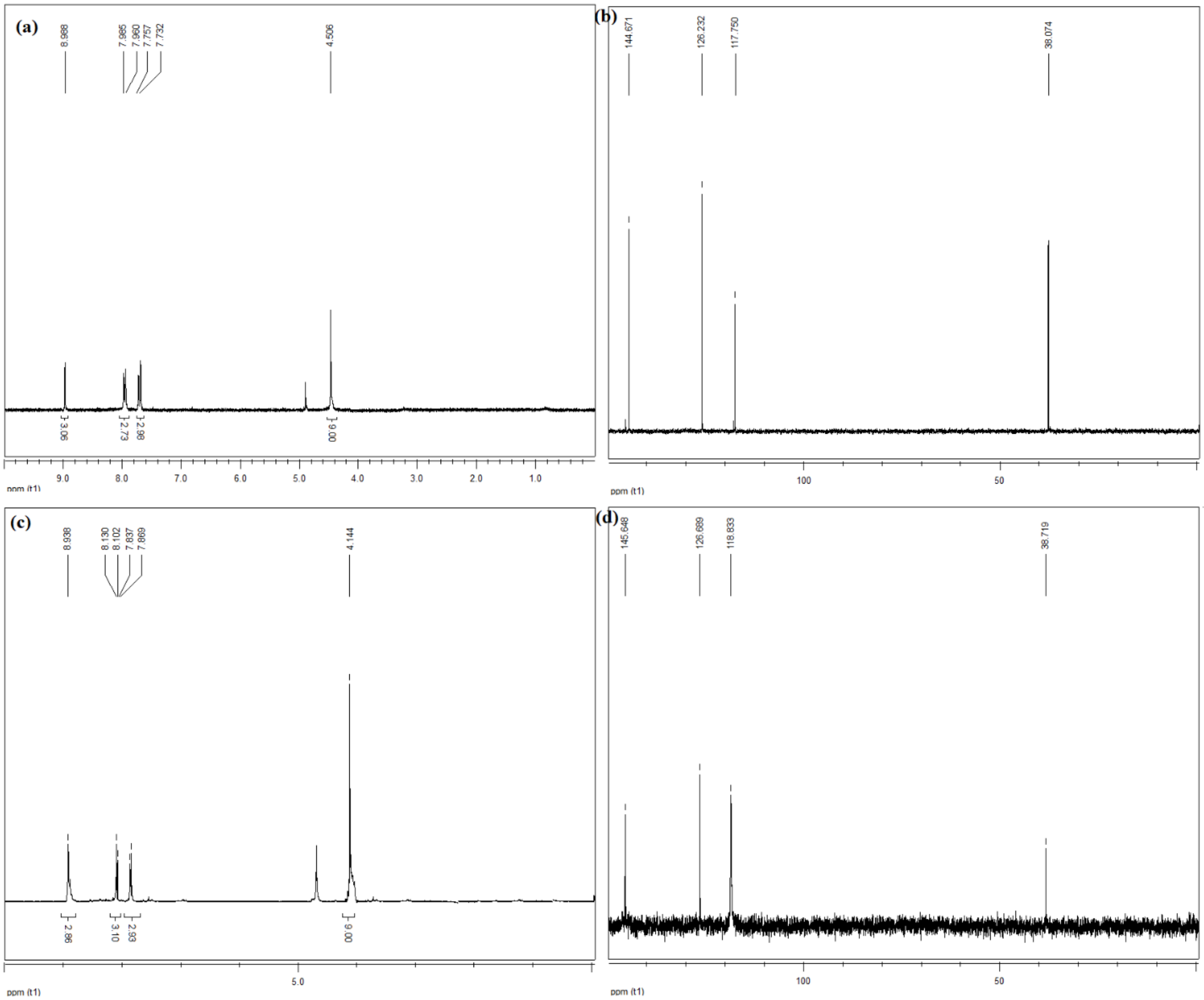

Fig. S2 ^{1}H-NMR (250 MHz, D_2O) spectra of (a) Im[I]TA **2**, and (c) Im[OH]TA **3**. ^{13}C-NMR (62.9 MHz, D_2O) spectra of (b) Im[I]TA **2**, and (d) Im[OH]TA **3**.

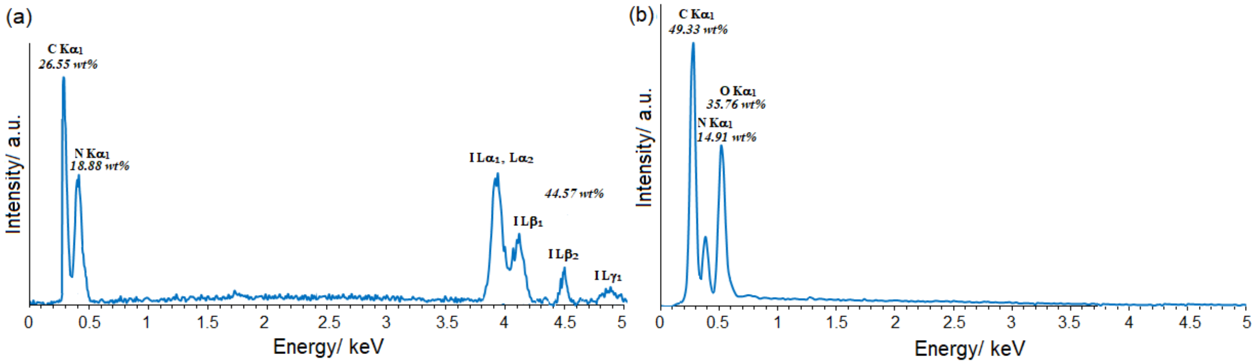

Fig. S3 EDX spectra of (a) Im[I]TA **2**, and (c) Im[OH]TA **3**.

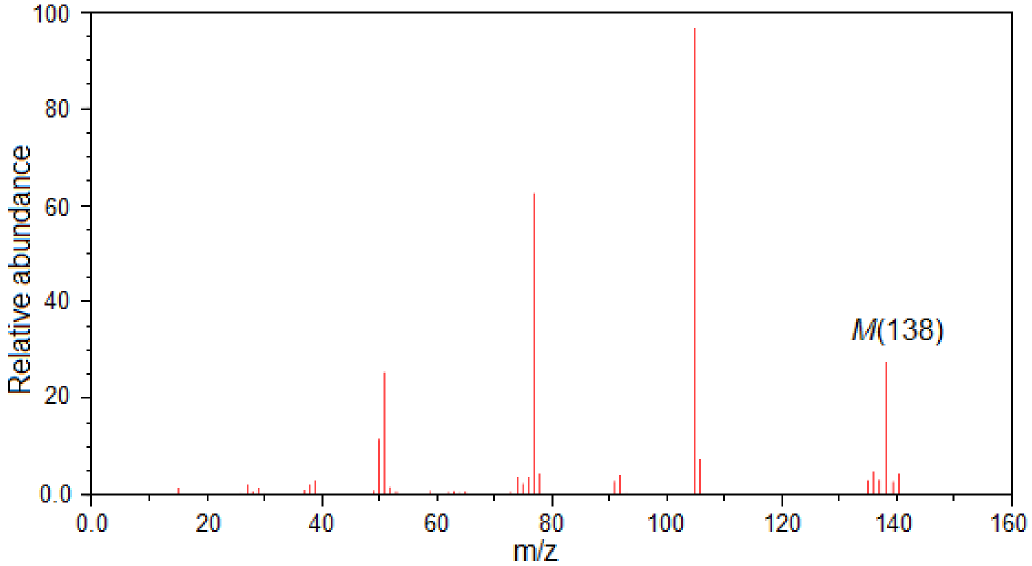

Fig. S4 Mass spectrum of ^{18}O enriched methyl benzoate

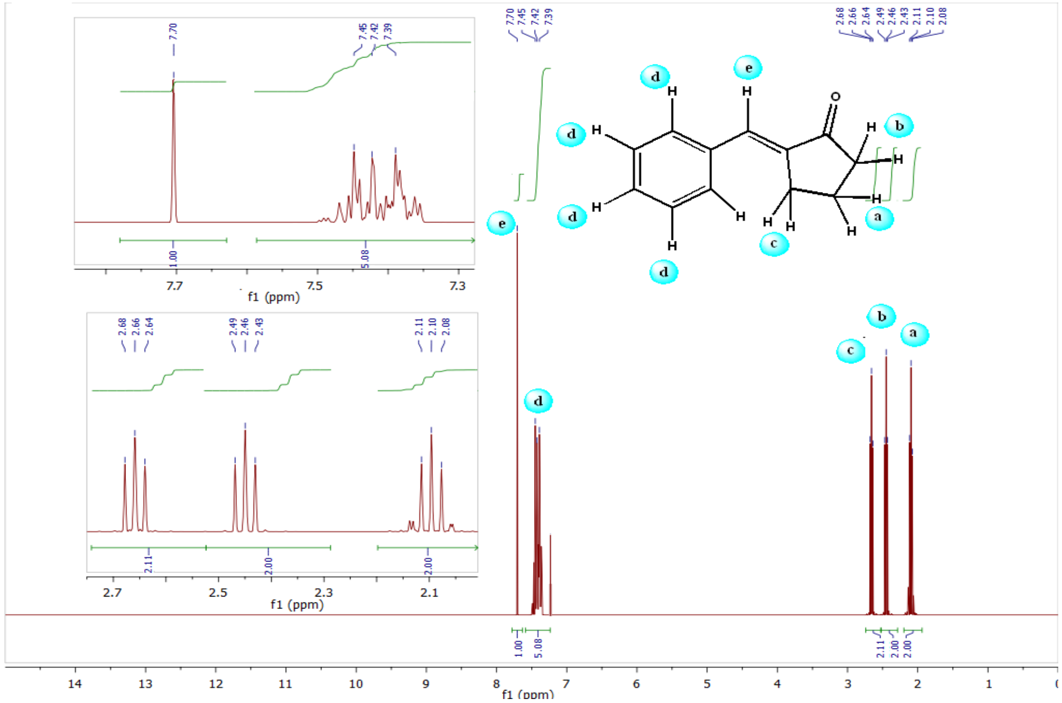

Fig. S5 ^{1}H-NMR spectrum of **7a** in $CDCl_3$ (250 MHz)

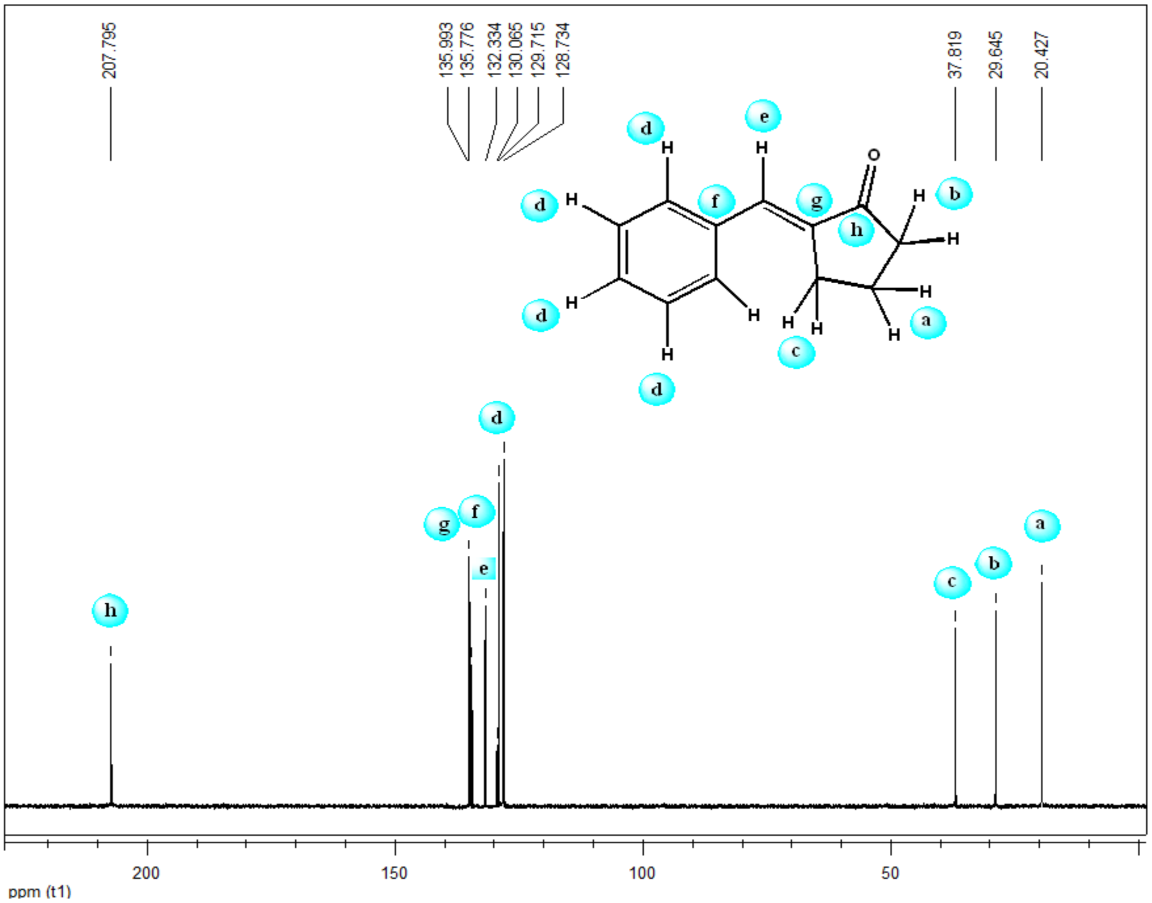

Fig. S6 ^{13}C-NMR spectrum of **7a** in $CDCl_3$ (62.9 MHz)

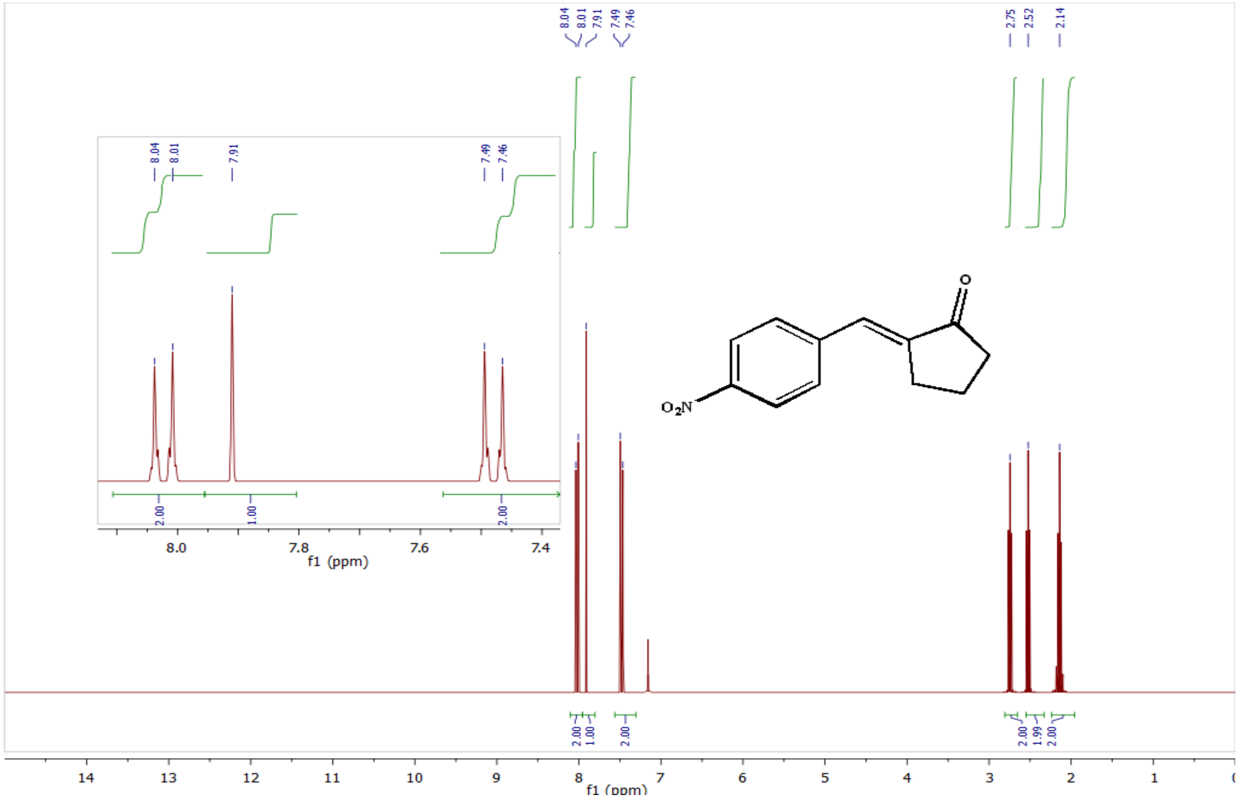

Fig. S7 ^{1}H-NMR spectrum of **7c** in $CDCl_3$ (250 MHz)

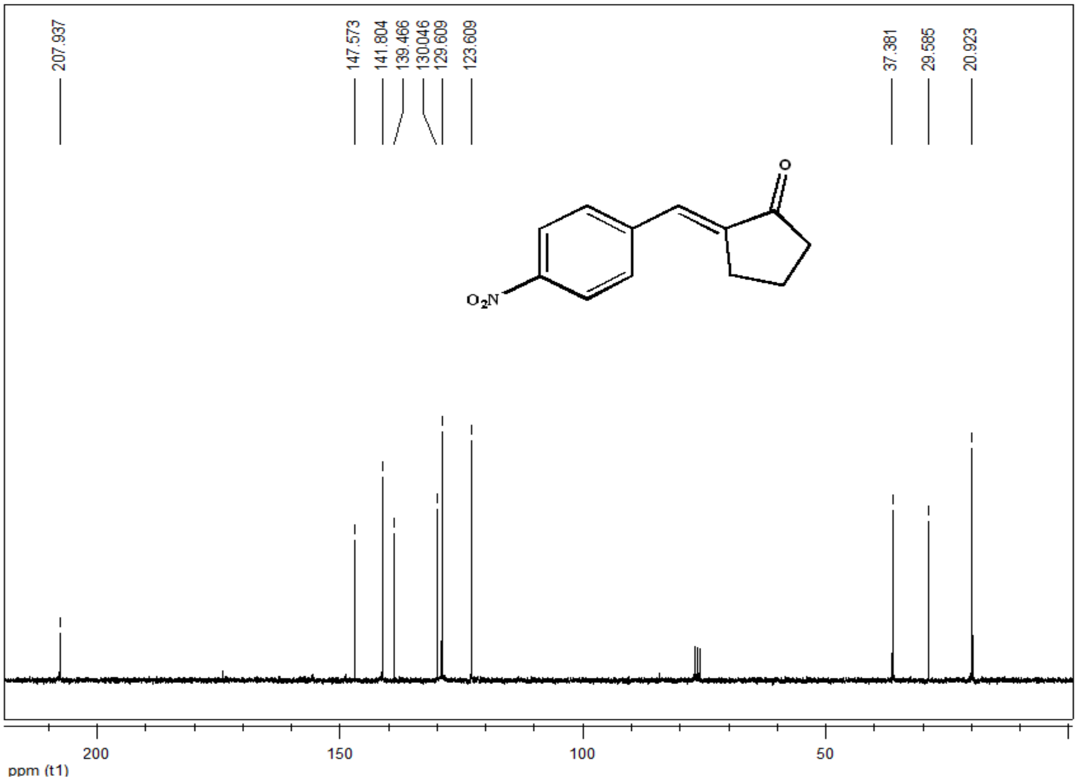

Fig. S8 ^{13}C-NMR spectrum of **7c** in $CDCl_3$ (62.9 MHz)

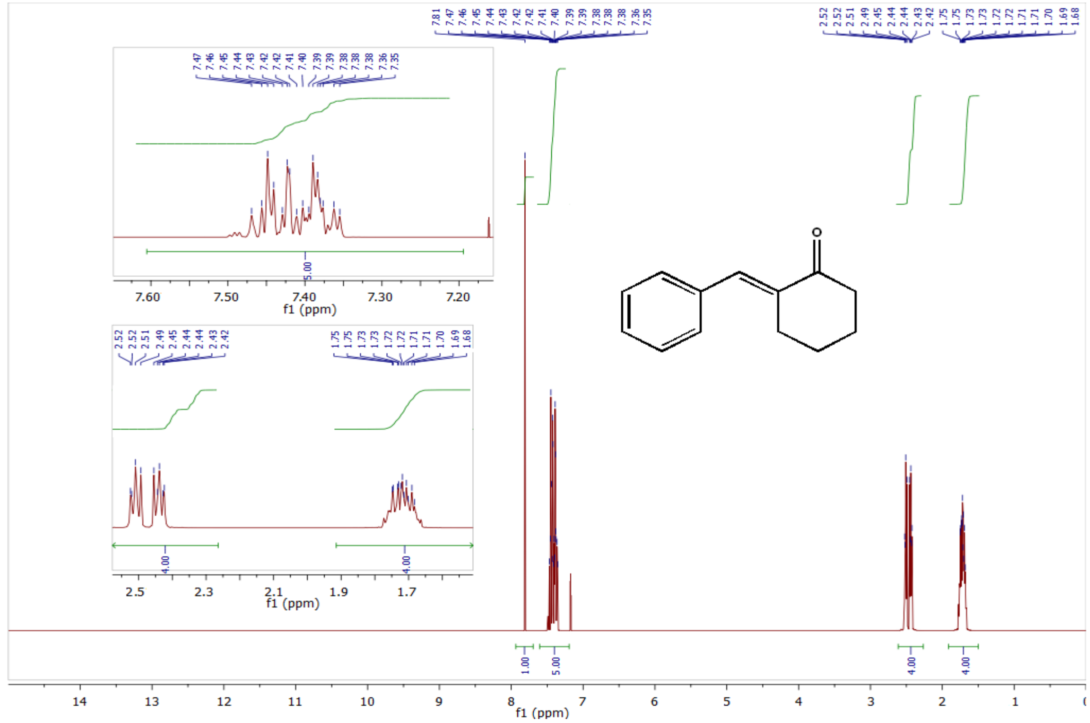

Fig. S9 ^{1}H-NMR spectrum of **7f** in $CDCl_3$ (250 MHz)

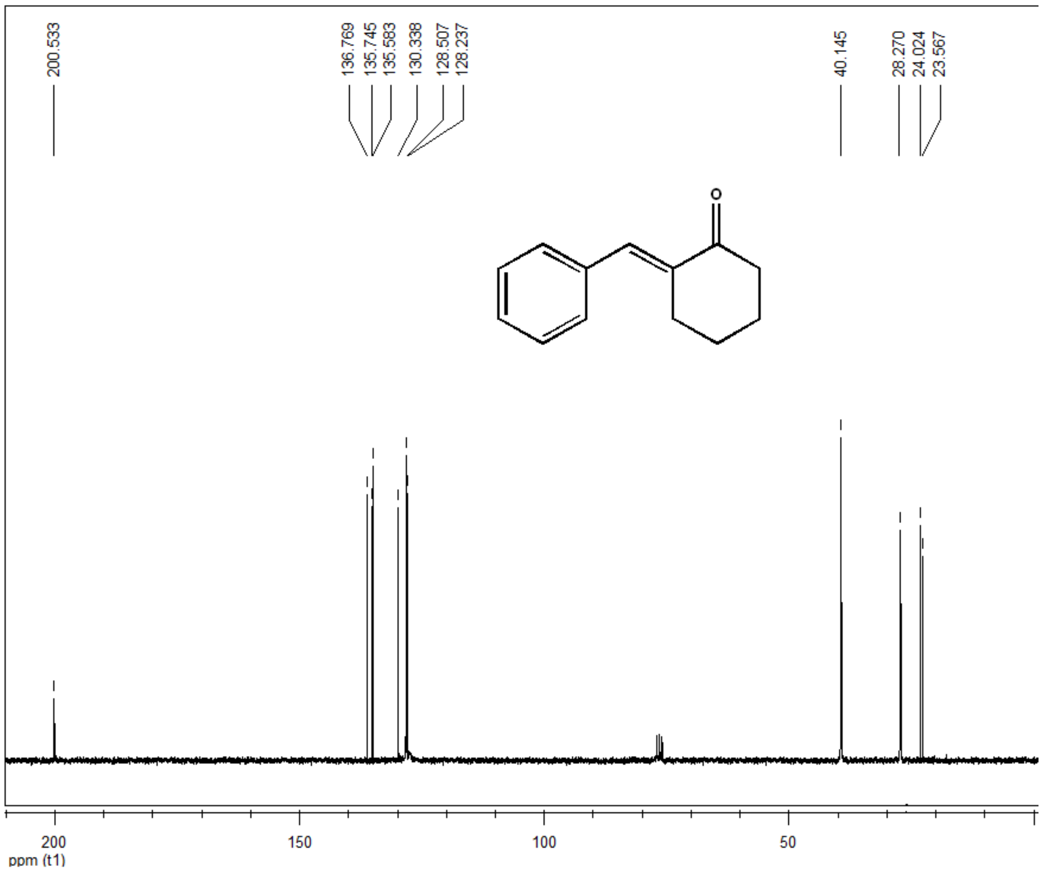

Fig. S10 ^{13}C-NMR spectrum of **7f** in $CDCl_3$ (62.9 MHz)

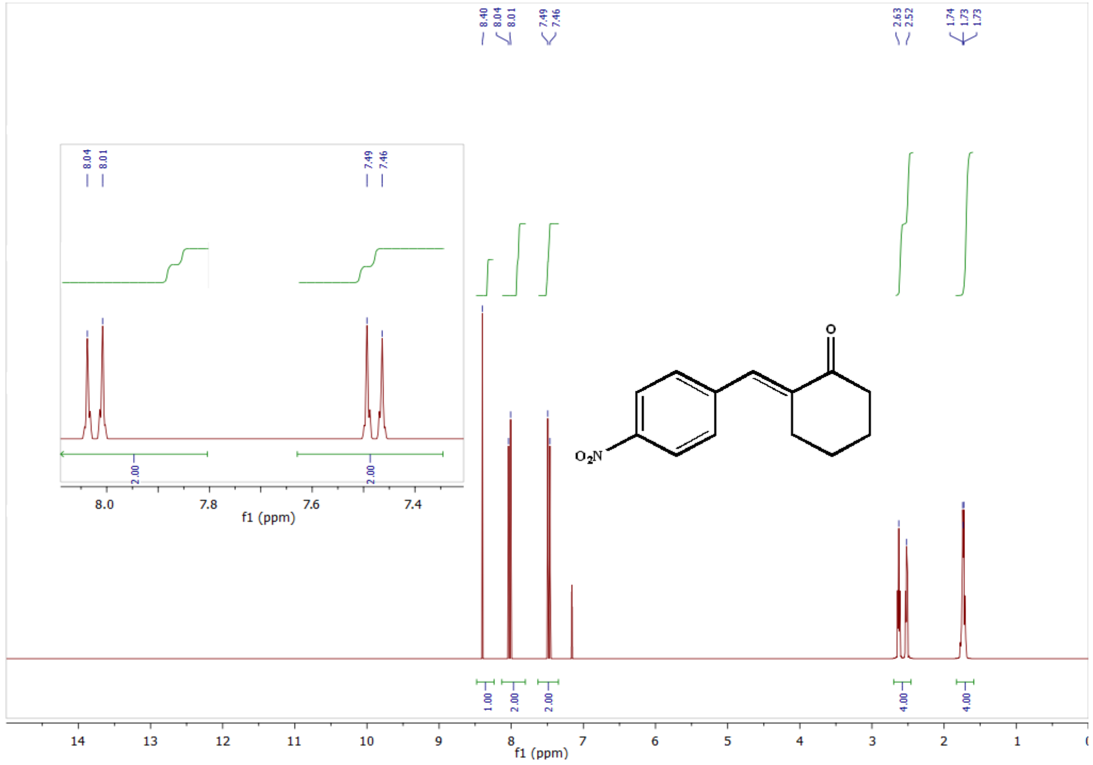

Fig. S11 ^{1}H-NMR spectrum of **7h** in $CDCl_3$ (250 MHz)

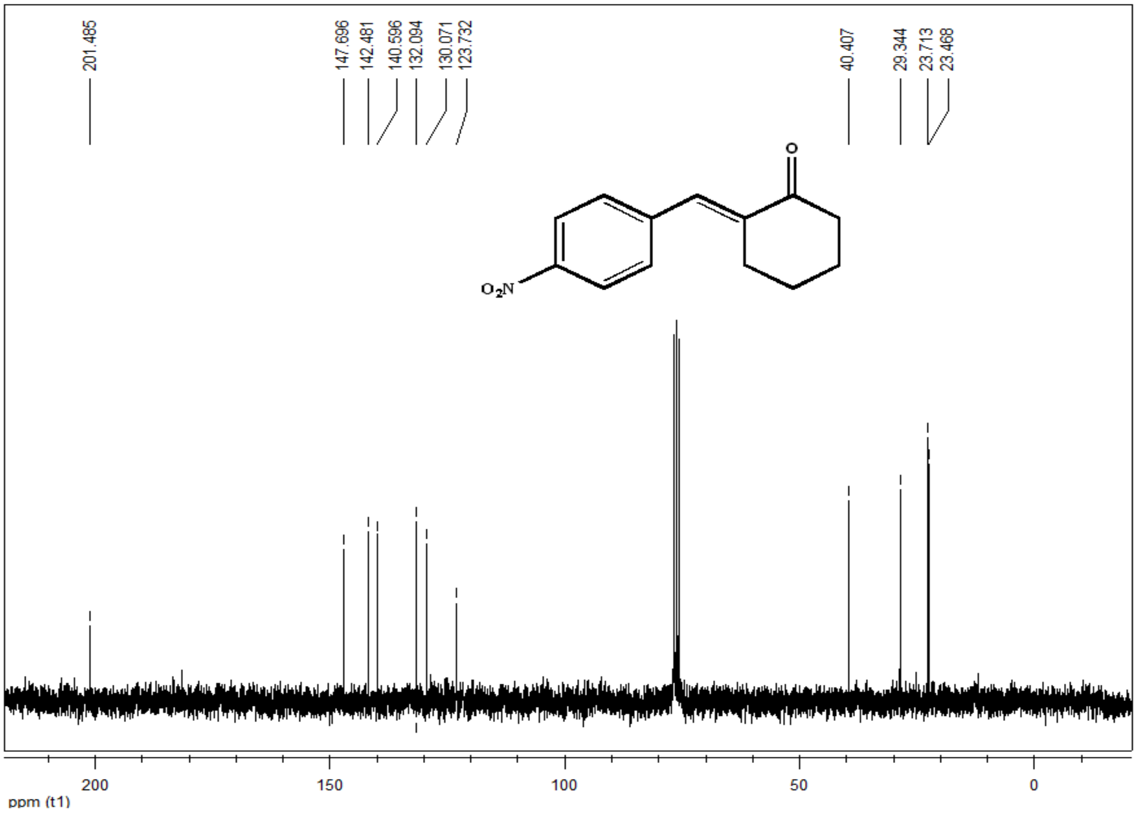

Fig. S12 ^{13}C-NMR spectrum of **7h** in $CDCl_3$ (62.9 MHz)
